# Proposal of a new shared payment model for healthcare financing in the United States: a hypothesis

**DOI:** 10.1186/s13037-020-00242-w

**Published:** 2020-04-23

**Authors:** Bruce H. Ziran, E. Kathleen O’Pry

**Affiliations:** 1 Hughston Orthopaedic Trauma, Atlanta, Georgia; 2 Philadelphia College of Osteopathic Medicine, Atlanta, Georgia

## Abstract

The healthcare repayment system in America is highly flawed due to several factors such as uncontrolled costs, unequal access, varied reimbursement systems, and complex patient interfaces. In fact, it is rated the worst among the eleven developed nations analyzed in the Commonwealth Fund’s evaluation conducted every three years. We propose a novel three-tiered model for healthcare repayment designed to fulfill the needs of the patients, the providers, the payers and the nation as a whole. We hypothesized that our new plan may spread cost between multiple entities and offer better coverage and access to care. Our model uses a shared-cost approach wherein the total risk expenditure becomes the responsibility of various stakeholders including the government, insurance industry, hospitals, patients, providers as well as the nation’s economy. While there is no perfect solution to healthcare in America, we believe our three-tiered model can create an economically balanced solution to break deadlock between party lines and result in better outcomes and patient care.

## Background

Healthcare spending in the United States (US) continues to grow and outpace inflation and other economic indicators. Per capita spending is estimated to exceed $10,000 and continuing to grow despite efforts to both slow growth and implement a risk-based system. Bundled care is one of the many solutions to mitigate this problem but may have some unintended consequences which produce conflicts of interest and may even adversely affect the care provided [1, 2]. In the article by Hoff et al. in Health Affairs, a healthcare economist outlines the potential conflicts of interest noticed when there is only a fixed reimbursement utilized with multiple providers competing for the funds. In this model, the hospital wants to minimize a patient’s length of stay, the rehabilitation center wants to expedite discharge, and other providers appear to compete for the “bundle” of payment [3]. This model was intended to induce providers and stakeholders to cooperate with one another, but instead, it may induce providers to compete for funds, marginalizing the patient without an advocate to act in their best interest. The case presented by Hoff outlines the practical likelihood of such untoward consequences. It may be an example of how efforts that attempt to limit or reduce cost, as opposed to induce a shared contribution, may not always work as intended.

As of 2017 the payment of healthcare services in the US is conducted through one of several routes. Medicare provides for the elderly and those with permanent disability who require ongoing medical care (19%); Medicaid covers those of lower income as well as mothers and children via Federally subsidized state Medicaid plans and CHIP/WIC plans (15%), and then there are the various factions of Private Health Insurance which make up the majority of healthcare services (31%) and provide health insurance to both individuals and companies. Private pay or self-pay is less common, but still accounts for just over 10% of expenditures (See Table 1). Since 3rd party and out of pocket expenditures can coincide with private and government programs, the basis of estimating the costs of our proposed model will utilize expenditures and per capita costs of private and government expenditures as a worst-case scenario of spending.

**Table 1:** Payment of Healthcare Services in the U.S.

2017 Healthcare Expenditures	Total Expenditure ($billions)	Per Capita Expenditure	Covered lives (millions of persons)
Private	1200	$6040	200
Medicare	720	$12,456	58
Medicaid	586	$8103	72
3rd Party	358		
Out of Pocket (Self)	365		
Other govt (WIC/CHIP)	138		
Investment	163		
Total	3530		

One of the goals of the Affordable Care Act (ACA) was to distribute risks and costs by reducing government spending and induce younger, healthier patients, to buy health insurance from government exchanges. By subsidizing payments for those who are unable to afford the full cost of health care, the ACA worked indirectly to help alleviate the costs of Medicaid [2]. While the ACA helped many obtain insurance, there are others who were either priced out of the market or obtained insurance without access to private providers who do not accept government plans [3, 4]. This is mostly because the insurance options available through the healthcare exchange are essentially Medicaid products which typically have low reimbursements for providers.

The shift in cost has forced plans (both individual government options and those which are employer provided) to incur very high deductibles and/or co-pays, which subsequently reduces the ability of lower income patients from either purchasing or using their health insurance. Furthermore, the affordability of the premiums and out-of-pocket expenses of the ACA requires patients to participate with discerning healthcare decisions and, as a result, patients may choose to not seek healthcare due to the anticipation out of pocket costs [5]. While involving patients in their healthcare decisions is a desired goal of any healthcare model, the complexities of understanding the financial aspects of their plan may ultimately result in a patient populace making less proactive medical decisions and instead having more reactive healthcare treatments.

There is a current effort by some advocates to move towards a single payer system in the US but there are several obstacles that would make such a plan unfeasible. First, a very strong insurance lobby would be against such a system as it would reduce the insurance market [6]. Second, even if the average per capita cost for a single “Medicare for all” system was midway between (~$10,000) the per capita cost of Medicare ($12,456) and Medicaid ($8103) the cost of government sponsored insurance would subsequently rise from around $1.3 trillion to over $3.1 trillion per year ($10 k per population of 300 m) [1]. Some estimate this cost to be even much higher. To fund this system entirely by the government, massive tax increases would be required which could offset any potential economic benefits [7]. Another obstacle “Medicare for all” would face is the lack of any competition, or potential accountability, as has been seen in the archetypal government system - the Veteran’s Affairs (VA) hospitals. Despite polls indicating a preference for a universal healthcare system, those numbers decrease significantly when posed with the financial implications and potential quality issues of such a plan [8].

Finally, continuing with a fee for service system would continue the historic growth of spending which is a national fiscal challenge and unsustainable. Other groups have advocated a free market approach where consumers (patients) use Health Savings Account (HSA), and a high deductible plan that would allow consumers greater flexibility in their decisions. While this system has virtue in free market principles, there are impediments due to the complexity of the healthcare system and the numerous options and decisions required by patients, who for the most part may not be able to navigate the complex financial aspects of such a model.

Despite all of the outlined issues, we continue with our current system where most healthcare premium costs are borne by one entity with ever increasing burdens on the beneficiary. Most efforts at cost reduction focus on using “shared risk” models however, the counterintuitive, and logical alternative of layered cost sharing of healthcare has not yet been considered. Currently, we are not aware of any efforts describing a “shared cost” model. The present paper presents a concept that proposes a shared cost method of funding healthcare that would be fiscally attainable, would provide comprehensive healthcare to the nation, and would benefit each stakeholder.

## The hypothesis

### The economic model

Under the current system, the ACA may provide insurance, but it does not provide access to many of the private specialties [9]. Essentially, the ACA provides *universal access*, but without *equal access*, with pricing challenges that prevent enrollment by many. Therefore, while many have “insurance”, that insurance is not accepted by many providers because it is essentially Medicaid. Alternatively, if we had a Medicare for all model, more providers would participate but even Medicare may not be sufficient to cover the costs of providing healthcare for many institutions and practices. Even so, due to the estimated costs noted previously, it too, would likely face great opposition.

Our proposed plan addresses four contemporary issues: 1) a basic insurance plan for all provided at no cost to the patient; 2) access to cost appropriate healthcare options; and 3) a sustainable method of funding; 4) benefits to each stakeholder. The plan would consist of a *three-tiered plan* of healthcare options that can be stacked or layered for benefits, and whose costs would also be shared by various funding entities. It would allow insurance companies to administer and compete in the market place. It would reduce costs for employer sponsored health benefits. It would also guarantee healthcare coverage for the poor and some form of payment for all providers.

In the first tier, every citizen (and qualified resident) would be provided basic health insurance which would be similar to existing Medicaid. This health insurance would be *funded by the government but administered by private insurance companies*. The entire current cost of Medicare and Medicaid would be more than enough to provide this level of insurance with funds to spare. Since this is a “no cost and universal” option for patients, they would have to understand the limitations of such a plan and be willing to participate in the “public” healthcare sector. To continue elderly and disabled citizens’ plans that would emulate current Medicare, slight increases in funding would be necessary and could be achieved through a small tax increase (e.g. payroll deductions). However, the increase would be far less than any tax required for a Medicare for all system. (see below).

In the second and third Tiers, insurance options for purchase would be analogous to the HMO, PPO, POS plans. The cost of these levels of coverage would be shared by using Tier 1 financing by the government (as a subsidy or voucher) and supplemented by employer or individual financing. Simply put, the government provides funding to either obtain Tier 1 coverage at no cost, or to use Tier 1 funding (voucher or subsidy) to apply towards Tier 2/3 coverage.

The Tier 1 plan would be a national entitlement and serve as a safety net for those without income or those who choose not to pay for a higher level of healthcare. The basic government plan would guarantee access to all public sector programs and private sector programs that opt to participate. Essentially, this basic “universal” healthcare plan would take its place among other governmental entitlements such as housing, food, childcare, etc. Most importantly, it would be transportable across all states and would not exclude pre-existing conditions.

With our model the government would no longer be an “administrator” of healthcare but instead would transition into a “financier” of healthcare. A second major advantage of this model is for the employer/business sponsored healthcare plans. Since the government would provide the first layer of payment, the employers cost of providing more comprehensive healthcare plans (HMO, PPO, POS) to their employees would be reduced since the employer would only need to provide the financing to purchase the next tier of benefits, done by paying the difference between tiers. They would use a subsidy or voucher from Tier 1 cost and apply those funds to purchase Tier 2/3 plans. Ultimately, the public would need to accept a tiered system and not consider it as exclusionary or divisive. While some might think such a tiered system “stratifies” healthcare, the two current alternatives (the present system and Medicare for all) also have a tiered access that creates strata by level of enrollment and provider participation. In reality the current system is already a tiered system, though it is not labelled as such.

Funding of this plan would require rethinking how healthcare is financed and would necessitate collaboration amongst the government, insurance industry, and employers that pay for employee healthcare. Yet, the plan would satisfy the demands of the public, the challenges of government spending, the concerns of the providers, and the interests of the insurance industry. The government would need to accept their role as financiers of basic healthcare without the need to administer the plan through either a federal or state-based system (Medicare and Medicaid). Not administrating healthcare would in and of itself save the government much needed funds since the “cost” of running Medicare/Medicaid is estimated to be approximately 2% of total expenditures. While private insurance administrative costs are estimated to be greater than 12%, the government would not bear that burden and would only be tasked with imposing regulatory boundaries [10].

The insurance industry would also need to restructure their payment models into a tiered ledger system that obtains its funding from multiple sources (government, employers, and individuals). Insurance companies would receive premium payments from the government for Tier 1 level services, and supplemental payments (from employers/ consumers) for Tier 2 and Tier 3 level services. Provider payments would be a summation of the reimbursement structures of each Tier plan. Since the government and insurance industry (employers and consumers) are not paying for plans that bear the entire cost of reimbursement, premium costs would decrease. This would thereby reduce their individual costs of operation as the essential tenet of the model is no single entity would bear the “entire” cost of services.

We can further elucidate our tiered cost sharing model with the following theoretical example. Let us assume that insurance premiums would be $800 per month for a Medicaid equivalent, $1600 per month for an HMO or PPO service, and $2400 per month for a POS plan. Currently, when an individual or employer decides to obtain or provide health insurance as a benefit, the majority of the premium is borne by the individual or employer. So, an employer would have to pay $1600 per month per employee for an HMO plan. In our model, employers have a government financed $800 per month per employee (the cost of Tier 1 plans) that can be applied to obtain Tier 2 coverage. In this scenario, an employer could provide a Tier 2 HMO plan to their employees at a cost of only $800 per month, instead of $1600 per month, since the government would be paying the first part ($800) of the Tier 2 premium. Premium costs are thus shared between the government and the employer/individual to obtain the secondary insurance tier. The same process would apply to get Tier 3 coverage, again provided as a benefit by the employer or purchased individually by the employee. Since that additional premium payment is just the differential cost between Tier 2 and Tier 3 premium costs, the individual would be more likely to pay for it themselves. Essentially, this stratified premium funding is shared by the national taxpayer base (the basic government plan), and the employer/business, and the individual who all pay premiums to purchase subsequent tiers of coverage.

One potential criticism of this model might be that if Medicaid was available to everyone, there would no longer be a need for employers to provide any healthcare above the consumer’s governmental Tier 1 plan. This could force hospitals and providers into delivering care to a large number of Tier 1 individuals, effectively being forced into a Medicaid healthcare system. This scenario can and must be avoided by using existing regulations which state that businesses of certain sizes must provide baseline second-tier healthcare options for their employees. However, since businesses would not have to pay for the entire cost of an insurance premium as they currently do, they would only have to pay for the Tier 2 or 3 portion of the insurance, which is the cost difference between Tier 1 (government subsidized) and Tier2/3 plan premiums. Not only would this be an incentive for business to provide healthcare benefits, but this reduced cost of providing healthcare would free up capital for businesses that could either stimulate economic growth, potentially creating more jobs, or induce more businesses to provide coverage for a greater number of their employees. Just like current ACA mandates on provisions of insurance, there would be safe guards to prevent a “Medicaid” for all system.

In order to cover insurance for the elderly we would need to address Medicare differently. The proposed plan would not require the Medicare system to bear the entire burden of cost only the incremental difference between the “Medicaid” cost level and its current “Medicare” cost level. Medicare would essentially become a Tier 2 or Tier 3 plan. This incremental cost might incur additional funding sources, but the amount required would be immensely less than that required for the “Medicare for all” plan. Currently, the per-enrollee cost of Medicare is approximately $12,300 as compared to Medicaid which is approximately $8000 [1]. The difference of $4300 (roughly over a third of Medicare spending) would need to be supplemented in some fashion, most likely by the government as it is a promised and expected entitlement into which US citizens have paid. At an enrollee roster of 57 million, the additional cost would amount to approximately $250 billion if funded exclusively by the government. While a marginal increase in the Medicare payroll deduction could easily fund this extra cost, another method of funding could be a reasonable form of “means testing” for Medicare enrollees to establish an income based premium supplement. The exact source of funding would require public debate and development by the legislatures. Nonetheless, $250 billion is much less costly than the $3.1 trillion of a “Medicare for all” plan.

### Funding the plan

The combined expenditure of Medicare, Medicaid, and other government plans adds up to approximately $1.5 trillion dollars. If that expenditure were divided by the total eligible patient population (~ 320 million), the per capita expenditure would be approximately $5000 and approximately 60% of current Medicaid spending. This $5000 per capita spending could either be deployed to insurance administrators, to individuals (via tax subsidies), or to individual states as block grants letting the states supplement that reimbursement rate as they felt necessary. This would provide Tier 1 coverage for the entire population, as well as a substantial savings to government healthcare spending.

The per capita target of $8000 would require an additional $3000 per capita, or $900 billion of funding. This is much less expensive than a Medicare for all system ($12,500 per capita) which would require an additional $7000 per capita spending for an extra $2.1 trillion, or a total of $3.75 trillion USD. To provide that funding an additional public revenue would be required (on the order of around $3000 per capita). If the per capita target was more competitive, as it is with the private insurance market, and on the order of $6000, only $300 billion of additional revenue (~$100 per capita) would be required for a Medicaid for all plan. That would be achievable with an additional payroll tax of approximately $200 on 150 million taxpayers or $600 on 50 million taxpayers (the higher income earners). While some may object to additional taxes, a system that provides for all and breaks the political deadlock would certainly be most beneficial for the country.

Expenditures of private insurance currently stand at approximately $1.2 trillion, but that spending is for an entire episode of care. Medicare spending per capita is approximately $12,000 and private spending per capita is approximately $6000. If Medicare and private spending were combined (recognizing they serve different subsets of the population) we can then identify the available funds for Tier 2 coverage. With $9000 per capita as a benchmark spending goal for private insurance (only $1 K more than current Medicaid and $3 k more than private per capita spending) that 6 k per capita of private insurance expense would only need an additional $4 K per capita to provide Tier 2 coverage. Tier 1 funding would be apportioned to provide the first part of premiums for Tier 2 coverage. Thus, by using the available government funds provided for the initial Tier 1 plans, the supplemental cost of Tier 2 coverage would be reduced to 2/3 of the previous fee for service rates ($3 k per capita more than $6 K per capita private spending).

Expenditures for out-of-pocket insurance stand at approximately $365 billion, which translates to approximately $1.2 k spent per capita. This incremental spending could be amortized into a third-tier insurance plan available to be purchased by an individual or provided by an employer. Better still, this amount could be used to fund the additional $250 billion noted above for Medicare enrollees.

As we can see with the previous three tiers of coverage, the current per capita spending of $11 k per consumer could be stratified into three tiers:

- Tier 1 is a basic government plan accounting for $4-6 K per capita.
- Tier 2 is a private employer/individual plan accounting for $4-6 K per capita.
- Tier 3 is a private employer/individual plan accounting for approximately $1-2 k per capita.

Combinations of these tiers would result in per capita spending commensurate with what we see currently in the fragmented, pay-it-all system. The top level of benefits could be achieved for between $9-14 K per capita. All three tiers combined would result in a near revenue-neutral national economic plan which “shares” both costs and premiums among the stakeholders. If the consumers and the government were to decide that a $5 k per capita Medicaid subsidy was insufficient to allow an “acceptable” level of health care, increasing that amount would only require a modest revenue raising endeavor (i.e. tax increase) as described previously. This could also be accomplished by a modest “means based” program that would shift some costs to individuals or employers in higher income brackets.

## Breakdown by stakeholders

### The government

The government would finance a “universal” healthcare plan that would cover all eligible lives and achieve what many industrialized countries have already. While the maintenance cost of Medicare for the elderly would potentially increase marginally, that cost is relatively small compared to a Medicare for all plan. It is also more financially efficient than continuing with a fractured system which does not have a single regulatory body overseeing the provision of basic and fundamental insurance. Since Medicaid and Medicare spending per enrollee is significantly higher than private insurance spending ($8 k and $12 K compared to $6 K, respectively), there is potential for streamlining services that would remain as “basic” and “essential” while reducing some unnecessary provisions. The government thereby benefits by having a single per capita expense for each eligible enrollee.

The government would then “outsource” administration of the insurance, as they currently do at the state level, to provide tailored delivery to consumers as determined by local needs. Block grants to states would be a simple method of reducing some of the administrative burden and costs of universal government healthcare. The government would oversee the insurance industry to ensure compliance and administration of the plans as well as encourage competition for quality and efficiency.

### The insurance industry

The insurance industry remains a strong part of the US market, accounting for over a third of all healthcare spending and covering over half of the population. The proposed model would benefit the insurance industry by outsourcing all administration of healthcare to them. They would not only administer the private tier of insurance (purchased by employer or enrollee) but would also administer the government plans (Medicaid and Medicare). Since the insurance industry would remain regulated in their overhead costs and coverage guidelines (i.e. pre-existing conditions), they would still benefit as they currently do with administering private plans. Additionally, their costs would be reduced because the insurance companies would not be accountable for all the healthcare costs of each enrollee. By reducing costs, they would theoretically be able to provide second tier insurance coverage at a much cheaper rate, thus making it vastly more affordable for businesses and private individuals. The reduced cost of obtaining coverage would result in a greater number of enrollees being covered with a “better than Medicaid” plan. The insurance industry would benefit by being contracted for each tier of insurance coverage. Competition would be maintained amongst insurance companies to provide the best service.

### The hospital

The hospital accounts for approximately 40% of healthcare expenses. Uninsured and under-insured patients remain an issue for many institutions. Having a system that guarantees Medicaid for all as well as making second tier plans more affordable is likely to result in a greater number of adequately covered patients (Tier 2 and Tier 3 patients). Currently, the floor of coverage is “self-pay” whereas with universal coverage the floor would be Medicaid. This concept was part of the fundamental theoretical argument for the ACA which tried to “encourage” enrollees to obtain a Medicaid equivalent for their healthcare or pay a fine. The Supreme Court ultimately decided that the ACA amounted to a constitutionally valid tax and affirmed its legality. However, the lack of the expected robust enrollment has still left many with no insurance, simply choosing to pay the fine instead. Additionally, a tiered system would allow for “sharing” a stacked premium and would encourage employers who would otherwise not be able to afford providing insurance for their employees to now offer the second tier of insurance thereby increasing the number of “adequately funded” enrollees. The net result would be in an increased number of adequately insured patients for hospitals with tangible benefits for nearly all healthcare institutions.

### The consumer (patient)

The greatest benefit of the plan would be to the individual patient. Not only would each eligible resident have a safety net of free healthcare as a floor, but the other options and tiers available would provide additional options at a subsidize or shared cost. With the cost of second or third tier insurance not being entirely borne by just businesses or individuals the higher tier plans would be more affordable for both parties and, therefore, more likely to be purchased. The result would be a greater number of people being covered in something other than the basic plan and have access to private sector healthcare.

### The providers

With this model providers would be assured of some form of payment from every patient. Additionally, a regulated insurance industry would be easier to manage because the government would ensure that all insurers “play by the same rules”. This would thereby reduce many of the administrative burdens involved in dealing with billing and reimbursements from different insurers who use different rules.

### The economy and nation

The economy would ultimately benefit as better access to healthcare for citizens translates into a healthier populace and, eventually, less individual healthcare burdens for individuals and families. By not having to worry about healthcare, individuals and families would have more disposable income and, as noted with tax cuts, potentially infuse those savings into the economic engine of the nation. Additionally, the increased revenue to hospitals and providers would result in more healthcare jobs. The stability of the healthcare sector would improve as there would be less uncertainty about coverage for the citizenry of the nation. As the world’s most industrialized and prosperous nation, a national healthcare provision would have the US be competitive amongst the other industrialized nations with much less uncertainty.

## Implementation of the hypothesis

There have been several attempts to provide basic healthcare to the citizenry of the United States, but most have met with criticism from various stakeholders. Politicians are influenced by their constituents, industry lobbyists, and healthcare systems that are intertwined in economic and political relationships. While the US spends more on health care per capita than any other industrialized country, it ranks lower in many healthcare metrics. Aggregate metrics may not reflect actual delivered quality of care due to an amalgam of factors including individual lifestyle choices, limited access, intervention, and preventative measures and the simple fact that some people continue to make poor choices which then result in poor healthcare outcomes. With almost half of all Americans having some type of chronic disease, conditions like diabetes and heart disease have directly influenced the rising cost of medical care, with those two alone being responsible for 85% of healthcare costs in America [11, 12]. Due to this, the per capita cost of healthcare spending in the US remains disproportional and continues to rise. Proposals to rein in costs and provide a comprehensive universal access system (i.e. a single payer system) have legitimate criticisms. The goal of providing a universal healthcare system for every citizen that also allows access to every provider is not only elusive, it is an economic impossibility. An alternative of providing a logical system that is advantageous and fair for all stakeholders should be a reasonable option. Some compromises would include a tiered system, minor tax increases, and reasonable regulatory oversight.

As an example, consider other entitlements such as housing, clothing, food, and transportation, which have differing levels of product offerings but provide a minimum basic sustenance of food, shelter, and transportation. No reasonable politician or citizen would suggest that those on entitlement programs should be provided luxury items, but we could all agree that there should be a safety net which provides adequate basic needs for our fellow citizens who may be less fortunate or unable to move up in society. The same logic would apply to a tiered healthcare system which favors the maximum flexibility, choice and allows costs to be shared by three societal sectors.. Additionally, the tax increases required for this system would be nominal compared to the current proposals and regulatory oversight would be to avoid fraud and exploitation.

Some might opine that this system is analogous to countries such as the United Kingdom (UK) where there is a baseline National Health Service (NHS) with the option to purchase “private” insurance for those who choose to do so. The difference with our model is that the operation of each insurance product is not with the federal government, as it is with the UK, but remains managed by either the private sector or individual states and is only partially funded by the government. The government may establish “regulation” for deployment of the insurance plans, but each tier of insurance would be administered by companies facile with healthcare provisions and subject to the current 15% percent administrative costs [13]. The latitude of individuals and employers to choose from a market menu of insurance products and providers will help promote efficiency and quality. The example of a government run system is concerning to many, especially considering recent issues with the Veterans Administration which is considered the archetypal governmental system. Dividing the cost amongst stakeholders, retaining free market principles, and providing choice to the consumer and employer is what makes this model different than the other socialized systems in the world.

The proposed financial model would satisfy the requisite needs of universal access and coverage for all citizens as a social safety net, but would also provide choices at various levels, and distribute shared costs to government, insurers, and consumers. As a safety net for all residents, the first-tier governmental product (which would provide adequate basic healthcare coverage) would come at no cost for the consumer. While some critics may opine that this tiered system may be “elitist” or restrict low income patients from access to care, the current system does the same - not by a defined tier but by implicit exclusion. Furthermore politicians and advocates may tout the increased number of “covered lives” with the current healthcare system, but most if not all those consumers only have access to public systems (just as the proposed Tier 1 system would have) because the private or Tier 2/Tier 3 providers do not accept the current ACA products, which are essentially rebranded Medicaid. The greatest criticism of all may come from the those who opine “there is simply no fixing healthcare”. However, that simply cannot be the solution. With the economically balanced proposed model it could break the deadlock between party lines and, through compromise, get the country what its citizens need without bankrupting us.

## Conclusion

In conclusion, our proposal provides an alternative but fiscally logical option to provide adequate healthcare for the nation, with little additional cost, and using market competition to drive efficiency.

## Data Availability

Not applicable. For any references on material sources see ‘References’ section.

## Abbreviations

US: United States
CHIP: Children’s Health Insurance Program
WIC: Women, Infants and Children
ACA: Affordable Care Act
VA: Veteran’s Affairs
HSA: Health Savings Account
HMO: Health Maintenance Organization
PPO: Preferred Provider Organization
POS: Place of Service
UK: United Kingdom
NHS: National Health Service
